# Visual Impairment in Women with Turner Syndrome—A 49-Year Literature Review

**DOI:** 10.3390/jcm13185451

**Published:** 2024-09-13

**Authors:** Ewelina Soszka-Przepiera, Mariola Krzyścin, Monika Modrzejewska

**Affiliations:** 1 II Department of Ophthalmology, Pomeranian Medical University, Al. Powstańców Wlkp. 72, 70-111 Szczecin, Poland; 2 Pediatric, Adolescent Gynecology Clinic, Department of Gynecology, Endocrinology and Gynecological Oncology, Pomeranian Medical University in Szczecin, Unii Lubelskiej 1, 71-252 Szczecin, Poland

**Keywords:** Turner syndrome, ophthalmic disorders, ophthalmology, genetics, monosomy

## Abstract

**Aim:** Among the severe organ complications occurring in patients with Turner syndrome (TS), ophthalmic dysmorphia and visual impairment are usually marginalized. There are only a few studies that take into account the prevalence of ophthalmic disorders in female patients with TS. **Material and methods:** Articles in PubMed, Scholar, and Website were reviewed, considering the prevalence of various ocular disorders in patients with X chromosome deficiency. Current standards for the management of patients with TS in the context of the prevalence of ophthalmic disorders were also analyzed. **Results:** Identification of visual impairment in people is important because it significantly impairs quality of life (QoL) along with other health problems. QoL affects cognitive and behavioral functioning and significantly increases self-esteem, acceptance of treatment, and, consequently, physical and mental health. Low self-esteem makes patients feel helpless and unable to plan their social development. Patients with TS are relatively more frequently diagnosed with various eye defects compared to the healthy population. Therefore, special attention should be paid to the early assessment of the visual system in people with TS to eliminate any factors that could potentially impair their QoL. **Conclusions:** Patients with TS should be referred to specialist ophthalmologists, pediatricians, or optometrists for preventive care or early treatment of visual impairment. The authors point out the need for comprehensive ophthalmological examinations as standard management in patients with TS.

## 1. Introduction

Turner syndrome (TS) is a congenital genetic disorder occurring in the female sex with a characteristic clinical picture: low stature, sexual infantilism, a specific morphological phenotype, and the presence of a set of characteristic physical features associated with 45 X0 monosomy along with congenital malformations of internal organs [1]. The characteristic phenotype of the development of somatic and mental traits is associated with the presence of quantitative and/or structural aberrations of the X chromosome. The origins of TS are genetically distinct, with different karyotypes ranging from the well-studied 45,X monosomy to mosaicism (45,X/46,XX), the presence of isochromosomes (46,X,i(Xq)) or ring chromosomes (46,X,r(X)). Differing genetic patterns can affect the clinical picture of TS, including the incidence and severity of associated visual disturbances. The X chromosome carries several genes crucial to eye development. In TS, loss or alteration of these genes (such as SHOX, ZFX, and MRL) can lead to the development of visual disorders. The absence of a second X chromosome means that there is no backup to protect against the manifestation of the mutation that causes eye disease [2]. Additionally, the absence of a second X chromosome can alter normal epigenetic regulation, potentially leading to the dysregulation of X-chromosome-coupled genes important for eye development. Identifying the specific karyotype in patients with TS may be important for predicting the risk of visual impairment and guiding personalized care. For example, it may be that patients with a 45,X karyotype may require more intensive ophthalmologic monitoring [3]. Moreover, from the perspective of ophthalmic genetics in TS, the origin of the X chromosome (paternal or maternal) is important, with 60–80% coming from the child’s mother [4].

Among the various severe organ complications seen in this TS, ocular dysmorphia and visual impairment, despite their frequent occurrence, are marginalized. Few case series summaries assessing the prevalence of specific ocular disorders among patients with TS are found in the ophthalmic literature. The vast majority of the literature consists of single case reports or case series, from which it is difficult to draw firm conclusions for analysis [5]. A consensus working group (the European Society of Endocrinology and the Pediatric Endocrine Society, in collaboration with the European Society of Pediatric Endocrinology, the Society of Endocrinology, the European Society of Human Reproduction and Embryology, the American Society of Cardiology, the Society of Endocrinology, and the European Society of Cardiology) for patients with TS (at a consensus meeting held in Cincinnati, OH, USA, in July 2016) developed guidelines for the diagnostic management and treatment of systemic disorders. It was the first to highlight the need for prevention and early diagnosis, of ocular disease, with indications for management at specific age ranges [1]. Recommendations from other national scientific associations have also recently been published and have already drawn attention to the problem. Various care regimens for these patients are suggested, in which the role of the ophthalmologist is increasingly emphasized [6,7].

## 2. Methodology

**Figure 1:**
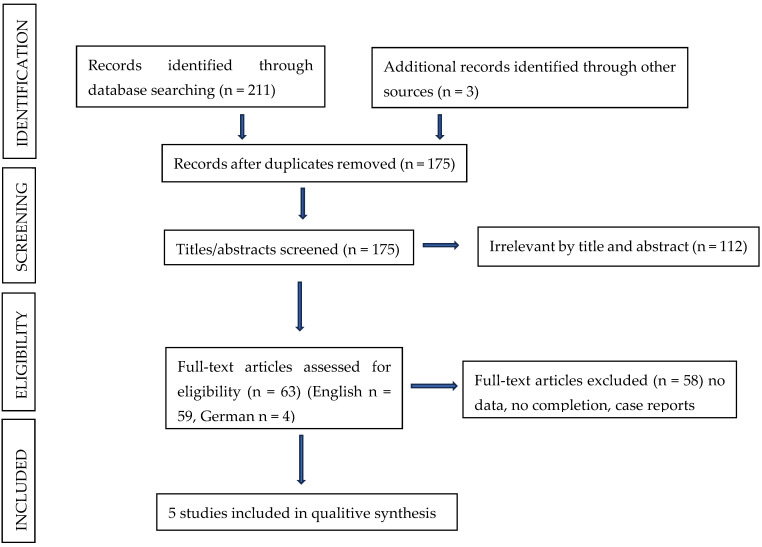
A flow diagram of the literature review process.

In May 2024, two authors (ESP and MK) independently conducted a literature search covering the period from January 1975 to April 2024. Three online databases were searched, PubMed, Scholar, and Website, identifying only clinical trials. Broad search phrases were used, which included the key phrases terms ‘Turner Syndrome’ or ‘Turners’ and ‘ophthalmology’, ‘ophthalmic’ or ‘ophthalmological disorders’. To obtain a broader search scope, a decision was made to include the terms ‘pediatric’ or ‘children’ in the search strategy. Duplicates identified during the initial search were removed first. The title, abstract, and keywords of the retrieved studies were then independently checked by ESP and MK. Any descriptions of single cases or clinical case series were discarded from the collection. Once potentially relevant studies were identified, full-text manuscripts were sought and then reviewed independently by ESP and MK. Only clinical studies evaluating the prevalence of specific visual defects in patients with Turner syndrome were selected for review. All studies that met the above eligibility criteria were included. Any disagreements were reviewed by a third investigator (MM) to reach a consensus. After applying the above criteria, 5 articles were included in this review. The literature review process is shown schematically in Figure 1.

## 3. Results

Table 1 summarizes the prevalence of various ocular abnormalities in Turner syndrome (TS) patients across several studies. Strabismus, refractive errors like myopia and hyperopia, and amblyopia are common, with prevalence rates varying widely between studies. Other conditions, such as ptosis, epicanthus, and nystagmus, are also observed but with less consistency across different cohorts.

## 4. Discussion

**Strabismus.** Strabismus is found in 30–33% of women with TS (Table 2), while in the general population, the prevalence of this defect is between 2 and 5%. Exotropia is found in 2.1% and esotropia occurs in 1.2% of the population between the ages of 4 and 74 years. This difference is due to the higher prevalence of exophoria in the population between 55 and 75 years of age [11,12]. According to the Polish Ophthalmological Society 2021, strabismus in children is diagnosed with a frequency of 1.3 to 5.7%, depending on age [13]. The consequences of untreated strabismus can include visual impairment, stereoscopic vision, asthenopia, double vision, nystagmus, and abnormal head and neck alignment. Early diagnosis and treatment of strabismus give a good prognosis. The treatment includes refractive correction, orthoptic exercises, occlusion of the better-seeing eye, use of topical medications, or surgical correction of the extraocular muscles [14]. 

Numerous ophthalmic conditions are present in TS such as strabismus and refractive errors such as varus, high bilateral refractive error, or visual deprivation, which is the cause of visual impairment occurring in 29% of this syndrome (Table 2). In comparison, in the general population, visual impairment occurs in between 1.75% and 3%, whereas severe visual impairment occurs in 1.2% of cases [15,16]. The prevalence of refractive errors in patients with TS, according to data obtained from various publications, is approximately 40%, with myopia increasing from 13% to 20% over 12 years. The prevalence of hyperopia over 10 years was estimated at 27% (Table 2). According to results from 15 European population-based cohorts and cross-sectional studies obtained between 1990 and 2013 published in European Eye Epidemiology, myopia was present in 30.6%, hyperopia in 25.2%, and astigmatism in 23.9% [17].

**Refractive errors:** Refractive errors are common vision problems that occur when the shape of the eye prevents light from focusing correctly on the retina. Meta-analyzed data from European studies indicated age-standardized prevalence rates for myopia at 30.6%, hyperopia at 25.2%, and astigmatism at 23.9% [17].

Among refractive errors, high myopia is the biggest problem and is associated with complications such as macular degeneration, retinal detachment, cataracts, or open-angle glaucoma [18]. These complications can lead to irreversible visual impairment in later life [19]. No less of a problem is inadequately treated hyperopia, the complications of which can lead to visual impairment and strabismus [20].

Hormonal deficits in TS may also impact ocular disorders, e.g., exacerbating or destabilizing refractive errors. Some of the reports in the literature confirm the effect of low concentrations of sex hormones on corneal hydration, shape, and refraction [21,22,23].

**Ptosis:** Eyelid drooping (ptosis) is a common clinical sign encountered in TS, which can affect people of all ages (both neonates and the elderly). Drooping of the upper eyelid is accompanied by a narrowing of the vertical dimension of the eyelid stroma. Ptosis develops as a result of dysfunction in the muscles responsible for lifting the eyelid and/or the nerves supplying these muscles. Two separate muscles are involved in eyelid lifting: the upper eyelid lift muscle, innervated by the superior branch of the oculomotor nerve (n. III), and the superior disk muscle (Müller muscle), innervated by sympathetic fibers of the cervical ganglion, responsible for lifting the posterior part of the eyelid.

Under normal conditions, the upper eyelid is located 1–2 mm below the superior corneal stroma and the lower eyelid at the junction of the sclera and cornea. In some cases, overexposed ptosis due to pupillary obscuration can result in visual impairment or strabismus. Congenital eyelid drooping is usually an isolated symptom; however, it occurs in many syndromes, but rarely as a pathognomonic feature for TS. According to the literature, ptosis is a common feature of Turner syndrome, Smith–Lemli–Optiz syndrome (15%), or Noonan syndrome (12%) [24,25]. In a screening study on a large Asian racial population by Hu et al., congenital eyelid drooping was found in 0.18% of 247,389 healthy individuals [26]. In children, few data suggest that the congenital type is most commonly observed. In a study by Griepentrog et al. [27], they diagnosed this type of drooping in 90% of 107 of all children studied, and El Essawy et al. [28] diagnosed it in 68% of 236 children, while Berry-Brincat and Willshaw found the presence of ptosis in 41% of 186 individuals (including 76 children) [29].

**Ophthalmic dysmorphia.** Eyelid crevices, an oblique, antimongoloid positioning of the eyelid crevices may occur individually, but there are no reports in the literature of the occurrence of this ophthalmic feature in healthy individuals. The most commonly described dysmorphia of this type, together with other craniofacial developmental abnormalities, is found in genetic disorders, e.g., RASopathies or others occurring as single case reports [30,31,32]. In TS, congenital positioning of the eyelid crevices occurs with a frequency of approximately 9% (Table 2). 

**Eyebrow fold (epicanthus).** Epicanthus is congenital and sometimes familial. It is seen in East Asian populations, and in Asia, the incidence varies between 40% and 90%. The diagonal fold is seen in 20–35% of patients with TS (Table 2), whereas in the European population, the diagonal fold is seen in only 2% to 5% of the general population. More often, the diagonal fold is observed in patients with Down syndrome, fetal alcohol syndrome, or other much rarer syndromes, i.e., Turner, Klinefelter, and Ehlers–Danlos. If it obscures part of the eyelid crevice, it may require surgical intervention [33,34].

**Hypertelorism (telecanthus)** is a symptom of many syndromes involving craniofacial dysmorphia, and in TS, it occurs with an incidence of approximately 10%. It is thought to be caused by abnormalities in embryonic facial development occurring in the 4–8 weeks of fetal life. Hypertelorism is most commonly associated with craniosynostosis syndromes, central facial cleft, and some other genetic syndromes. Pseudohypertelorism, or the appearance of widely spaced eyes, can be found when examining patients with features such as a flat nasal ridge, angular folds, exotropia, widely spaced eyebrows, or narrow eyelid crevices. Disorders in which telecanthus is seen include Down syndrome, Ehlers–Danlos syndrome, Klinefelter syndrome, fetal alcohol syndrome, Cri du Chat syndrome, Waardenburg syndrome, and Turner syndrome. The overall estimated prevalence of hypertelorism in individuals with TS is rare, with some sources estimating it at around 0.002% of births [5,35,36].

**Daltonism.** Red–green color vision abnormalities are a recessive trait coupled to the X chromosome and the most prevalent among these disorders. In Turner syndrome, abnormal color vision occurs with a frequency of approximately 8%. As in the general population, a 2% to 8% frequency is observed, mainly in males, and about 0.5% is observed in females (of which Europeans are the most common and Africans the least common). Color disorders result from mutations in genes encoding red or green [37]. Recessive diseases coupled to the X chromosome, e.g., daltonism, are typically present in males [5].

**Accommodative disorders.** In healthy adults, accommodative disorders most often begin after the age of 40 and progress until the age of 70, when the eye completely loses its ability to accommodate. In young adults, accommodative dysfunction may occur in uncorrected hyperopia or certain ophthalmic diseases, such as iritis; however, there are no data on the percentage of the above types of this dysfunction. In patients with Turner syndrome after 18 years of age, accommodative dysfunction is noted in about 40% of cases (Table 2), most likely related to the faster aging of the connective tissue. 

Five types of accommodation disorders have been classified in the literature: inefficient accommodation (NSA—accommodative infacility, inflexibility), insufficient accommodation NDA (accommodative insufficiency), accommodative fatigue (MA, accommodative fatigue), accommodative sluggishness (OA accommodative inertia), and accommodative paralysis (PA accommodative paralysis) [38,39,40]. Among the most common accommodative dysfunctions are the NSA and NDA types, which are accompanied by headache and visual acuity instability during reading, visual ambiguity, eyeball pain, burning eyelids, or photophobia [38]. There are no reports in the literature on the type of accommodation disorder present in Turner syndrome. There are also no data that show a breakdown of this disorder according to age (pediatric and adult population). 

In female patients with TS, convergence disorders are common, as they occur in about 40% of cases (Table 2). This dysfunction of binocular vision results in symptoms such as eye fatigue, blurred or double vision, headaches, and reading problems [41]. In the general population, the disorder has been reported to occur at varying rates from 1.7 to 33% [41,42,43]. This is due to the different measurement methods and diagnostic criteria adopted [41,42,43,44]. The prevalence of convergence disorders in the literature has been averaged to approximately 5% [41,42].

**Cataracts** are a common eye condition characterized by clouding of the lens, leading to hazy vision or blindness [45]. Pre-stage cataracts, also known as juvenile cataracts, occur before the age of 40 years and involve lens opacification [46]. While they are less common than age-related cataracts, they can significantly impact vision. The prevalence of these defects varies, ranging from approximately 0.63 per 10,000 to 9.74 per 10,000 children, with a median prevalence of 1.71 [47]. In patients with Turner syndrome, this dysfunction is rare, occurring in only 3% [5]. Approximately 50% of juvenile cataracts are caused by genetic mutations in genes related to lens structure or clarity [46]. Risk factors associated with pre-stage cataracts include diabetes, high myopia, atopic dermatitis smoking, and exposure to heavy metals [43]. In the literature, we find a case report that confirms the association of TS with early-onset cataracts [48]. 

Congenital or childhood-onset cataracts are rare in any population but are more frequent in the setting of Turner syndrome. The reported prevalence of cataracts in TS patients ranges from 13% to 33%, compared to a rate of 4% in the background population [49].

**Nystagmus.** In children, congenital nystagmus (INS) is a condition with a typical onset before 6 months of age, which may occur as idiopathic or associated with disorders in the form of retinal dystrophy, visual impairment, visual deprivation, or a range of neurological conditions [50,51]. The prevalence of INS is estimated to be 0.14% while the idiopathic form occurs in 0.019% of cases [52]. Areas of visual dysfunction affected by nystagmus include visual acuity [53], stereoscopic vision [54], and contrast sensitivity [55].

Nystagmus may be a symptom of an X-linked disorder caused by the loss of one of the sex chromosomes, specifically a change in the FRMD7 gene; this disorder does not usually affect female individuals. A single publication has been reported in the literature in which a 3-month-old girl with abnormal horizontal eye movements (anomalous horizontal eye movements) underwent a series of diagnostic tests, including a karyotype study that confirmed monosomy X. This demonstrates the possibility of coexistence of FRMD7 X—a chromosome coupled with nystagmus and TS [56]. The prevalence of nystagmus in Turner syndrome was estimated by Dennison et al. to be approximately 9%—according to the data from the study [5].

**The blue sclera** is caused by thinning and structural changes in the sclera, making the underlying dark choroidal membrane of the eye visible. It is also frequently seen in connective tissue disorders: congenital brittleness of the bone, Ehlers–Danlos syndrome, craniofacial dysostosis, and Marfan syndrome. Blue sclerae mainly occur as one of the symptoms of various genetic syndromes, less frequently from other causes including as a side effect of medication. A cross-sectional review of the literature presented the most comprehensive set of etiopathologies coexisting with blue sclerae, which included dozens of genetic syndromes and other disorders (including iron deficiency anemia, Caplan syndrome, hyperhomocysteinemia, and myasthenia gravis, in patients with AIDS) and a few pharmacologically induced cases [57,58]. Given the frequent occurrence of osteoporosis in TS, it can be suggested that the blue sclerae in this syndrome may be due to collagen disorders [59].

**Glaucoma.** Congenital glaucoma in patients with TS is rarely described, mainly as case reports, while in the only publication cited by the authors, it occurs with a frequency of approximately 1% [5]. Primary congenital glaucoma (PCG) in the general population of Western countries has been reported in 0.01 to 0.003% of cases. The incidence of PCG varies considerably among ethnic groups; moreover, the higher incidence in individual countries is associated with a higher incidence of marriage among related parents [60,61]. Mosaicism-associated GG is associated with anterior segment dysgenesis, which increases the possibility of congenital glaucoma in GG [62]. Another report showed an increase in central corneal thickness (CCT) in patients with IOP compared to controls. An increase in CCT overestimates intraocular pressure measurements which plays a role in the diagnosis and treatment of glaucoma [63].

**Others.** Individual reports in the literature concerning TS include the following disorders: conjunctival lymphoedema, corneal cone anterior segment dysgenesis, retinal neovascularization, presence of choroidal neovascular membrane, and retinal detachment [62,64,65,66,67,68]. Of other rare ocular lesions of the posterior segment, there are retinal telangiectasias in Coats’ disease, a type of retinal degeneration [69,70,71]. 

In the literature, there are isolated cases of uveitis (Uveitis) of the eye associated with ocular TI [72,73,74,75]. They concern both anterior uveitis and bilateral uveitis, and sympathetic ocular inflammation and punctate internal choroidopathy have also occurred. Some of the patients described had autoimmune diseases (Crohn’s disease, juvenile seronegative arthritis, or psoriasis) or only markers of autoimmune, asymptomatic diseases. Since patients with TS have twice the risk of autoimmune disease, this may contribute to uveitis [76].

Cases of limbal stem cell deficiency (LSCD) in TS have been reported in the literature; this condition is accompanied by epithelial defects, keratosis of the cornea, or chronic inflammation, which can result in pain, photophobia, and even loss of vision. To prevent the progression of LSCD, inflammation within the eye must be prevented [77].

In patients with TS, there is also an increased incidence of a corneal cone (ectasia), which can occur spontaneously but also after LASIK (Laser-Assisted In Situ Keratomileusis, local laser-assisted keratomileusis) procedures. Therefore, this procedure should be considered in cases of ZT [49].

As key findings after reviewing the literature above, it should be noted that, in TS patients, strabismus is significantly more common (30–33%) than in the general population (2–5%), posing a risk of severe complications if left untreated. Refractive errors, including myopia and hyperopia, occur at a comparable or slightly lower rate but may present earlier or more severely in TS due to hormonal factors. Amblyopia is more frequent in TS patients (4–29%) compared to the general population (1.75–3%), stressing the need for early intervention to prevent long-term visual impairment. Ptosis is also notably higher in TS (5–21%) versus the general population, where congenital ptosis is rare (0.18%), possibly due to muscular or neurological issues in TS. Nystagmus occurs more often in TS patients (4–9%) compared to the general population (0.14%), suggesting a genetic link. Although cataracts are less common in TS (3–6%), they tend to appear earlier, necessitating regular eye exams. Color vision deficiency, while comparable to the general population, is notable in TS due to its X-linked inheritance. Convergence disorders are significantly more prevalent in TS (40%), which may contribute to the higher rates of strabismus and amblyopia. Rare conditions like blue sclerae and glaucoma are also observed, possibly related to connective tissue disorders and ocular pressure issues in TS. Lastly, hypertelorism is more common in TS (10%) than in the general population, although it remains relatively rare overall. Ophthalmologic abnormalities are more frequent in TS patients than in the general population but are not specific: refractive anomalies, strabismus, orbitopalpebral malformations, etc. Patients are at risk of amblyopia during the sensitive period of visual development (first decade of life) [5,12]. Meta-analyses of the data indicate that 19% of patients with TS had ocular disorders compared with 3% of women in the general population (10).

The relationship between ocular disorders and the patient’s karyotype is complex. Research reveals mixed findings: some studies suggest that individuals with mosaic karyotypes, which involve multiple cell lineages, may have a higher risk of anterior segment dysgenesis (e.g., glaucoma or abnormal irises) due to potential neuronal migration defects [8]. However, other studies, including large cohorts, do not find a consistent association between specific karyotypes (like 45,X or mosaicism) and the prevalence of eye disorders such as strabismus, refractive errors, or cataracts [7,9]. This indicates that the prevalence of eye disorders in TS is not consistently associated with specific karyotypes, indicating that other factors may play a more significant role.

Identifying visual impairment in people is important because it significantly impairs quality of life (QoL). QoL affects cognitive and behavioral functioning, significantly increasing self-esteem, acceptance of treatment, and consequently physical and mental health [78,79]. Patients with TS often struggle with a variety of conditions, including those involving cognitive and behavioral dysfunction. Low self-esteem makes patients feel helpless and unable to plan their social development [80]. Therefore, early recognition and proper treatment of visual impairment in this group of patients appears to be exceptionally important.

Several guidelines for conducting proper care for patients with TS have already appeared in the literature. Some of them take into account the need for regular visual inspections. Below, in Table 2, we present our suggestions for the care of this group, mostly based on recommendations from the “French National Diagnosis and Care Protocol” according to Fiot et al. [6].

The strength of the manuscript is that it covers various ocular abnormalities in TS, drawing on multiple studies. It effectively compares ocular disorder prevalence in TS patients with the general population, emphasizing the higher risks in TS. We discuss the importance of early diagnosis and regular eye monitoring, underscoring its relevance to clinical practice. Finally, we introduce some guidelines for ophthalmic care in TS patients. However, some limitations need to be noted. Excluding case reports may overlook rare ocular conditions in TS patients. This review includes studies dating back to 1975, which may not align with current clinical practices due to advancements in the field. This article briefly mentions genetic factors but lacks an in-depth exploration of how genetic variability in TS influences ocular disorders.

## 5. Conclusions

Physicians treating patients with TS tend to focus on the large life-threatening defects of the syndrome, neglecting ophthalmological conditions. Of the numerous eye defects, the most important are those that lead to visual impairment and, if not recognized early, can result in visual impairment. Patients with TS must be referred to specialists (ophthalmologists, pediatricians, or optometrists) early to apply prophylaxis that protects against ocular complications. 

## Figures and Tables

**Table 1:** Ocular abnormalities present in patients with TS and their approximate prevalence compared to the general population based on data from the literature.

Series	Denniston and Butler [5]	Brunnerova and Lebl [8]	Wikiera et al. [9]	Viuff et al. [10]	Huang et al. [7]
Year	*2004*	*2014*	*2015*	*2021*	*2022*
N	274	81	82	1156	187
Ocular abnormalities	%	%	%	%	%
Epicanthus	35	20			2
Ptosis	21	5			2
Hypertelorism, wide orbital spacing	10				
Antimongoid positioning of the eyelid slits	9				1
Strabismus, strabismus	33	10		4	13
Phoria only	46				1
Exotropia	9		1		9
Esotropia	20		20		4
Hypertropia	3				
Refractive disorders	-	52			
Myopia, myopia	13	29	12		24
Hypermetropia, hyperopia	27	24	32		16
Astigmatism			17		14
Red/green color vision disorder	8	12	5	0	1
Accommodative disorders	40				
Convergence disorders	40				
Nystagmus	9		4		2
Cataract	3			6	
Amblyopia (strabismus, refractive errors: bifocality, or high bilateral refractive error, visual deprivation)	29	12	16		4
Glaucoma	1			1	
Blue sclerae	2				
Conjunctival disorders				2	
Disorders of the sclera, cornea, iris, and ciliary body				2	
Disorders of the choroid and retina				4	
Diseases of the vitreous body				29	
Disorders of the optical nerve				0.5	
Disorders of refraction and accommodation				3.46	

**Table 2:** Suggestions for ophthalmic subject and physical examination in patients with TS.

In the case of prenatal or perinatal diagnosis of TS, systematic ophthalmological examinations should be performed from 3 months of age (screening for factors that increase the risk of strabismus; strabismus and/or refractive anomalies; and/or organic abnormalities).
Otherwise, systematic ophthalmic examinations should be performed during the diagnostic evaluation. Patients should undergo an ophthalmological examination between 3 and 4 years of age, as in the general population. Patients should be checked for suspicious signs or characteristic clinical signs: Nystagmus/abnormal eye movements; Behavior suggesting visual impairment; Anatomical appearance of the eyes: white spot on or in the eye, eye is too big or too small; Pupils of different sizes; Drooping eyelids; Abnormal white, yellow, gray, or red reflections in the eyes; Eye deviation/pseudostrabismus/true strabismus. Suggested ophthalmic examination scheme: **1. General and family history** **2. Examination of the orbit and accessory organs of the eyeballs:** - palpation of the orbit - eyeball position - eyeball alignment - eye movements - eyelid crevice width - eyelid positioning - eyelash assessment **3. Anterior segment examination:** - conjunctiva - cornea - lens - corneo-ocular angle - assessment of the vitreous body **4. Anterior segment examination:** - conjunctiva - cornea - lens - corneo-ocular angle - assessment of the vitreous body **5. Posterior segment examination:** - fundus examination - macular function assessment - assessment of optic nerve function
Other specialized tests are required in the following cases: (1) ophthalmic abnormalities requiring specific and appropriate monitoring and management, the frequency of which is decided by the ophthalmologist; (2) the appearance of suspicious sensory or motor symptoms.
